# Supplementary material for: Sustainable Cellulose-Aluminum-Plastic Composites from Beverage Cartons Scraps and Recycled Polyethylene
Source: Polymers (Basel). 2022 Feb 19;14(4):807. doi: 10.3390/polym14040807 (PMC8963112; doi:10.3390/polym14040807)
Supplement: Supplementary file 1 [file polymers-14-00807-s001.zip › polymers-1501674-supplementary.pdf]

Supplementary material

Sustainable cellulose-aluminum-plastic composites from beverage cartons scraps and recycled polyethylene

Irene Bonadies [1], Roberta Capuano [1,2], Roberto Avolio [1,*], Rachele Castaldo [1], Mariacristina Cocca [1], Gennaro Gentile [1] and Maria Emanuela Errico [1]

[1] National Research Council of Italy, Institute for Polymers Composites and Biomaterials (IPCB-CNR), Via Campi Flegrei 34, 80078 Pozzuoli (NA), Italy; roberto.avolio@cnr.it (R.A.); irene.bonadies@cnr.it (I.B.); roberta.capuano@ipcb.cnr.it (R.Cap.); rachele.castaldo@ipcb.cnr.it (R.Cas.); cocca@ipcb.cnr.it (M.C.); gennaro.gentile@cnr.it (G.G.); mariaemanuela.errico@cnr.it (M.E.E.)

[2] Department of Mechanical and Industrial Engineering—DIMI, University of Brescia, Via Branze 38, 25121 Brescia, Italy

* Correspondence: roberto.avolio@cnr.it; (R.A.)

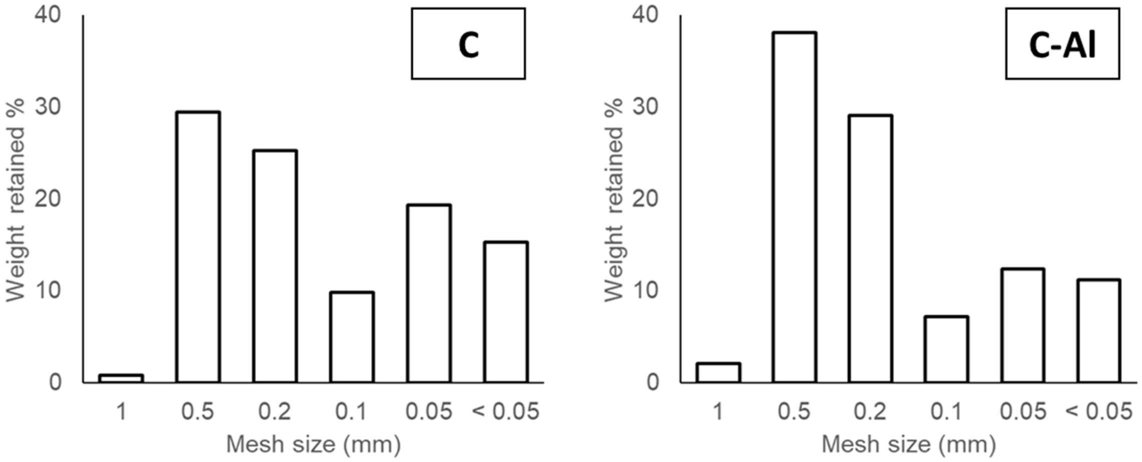

Figure S1. Particle size distribution of milled C and C-Al cartons as measured by sieving.

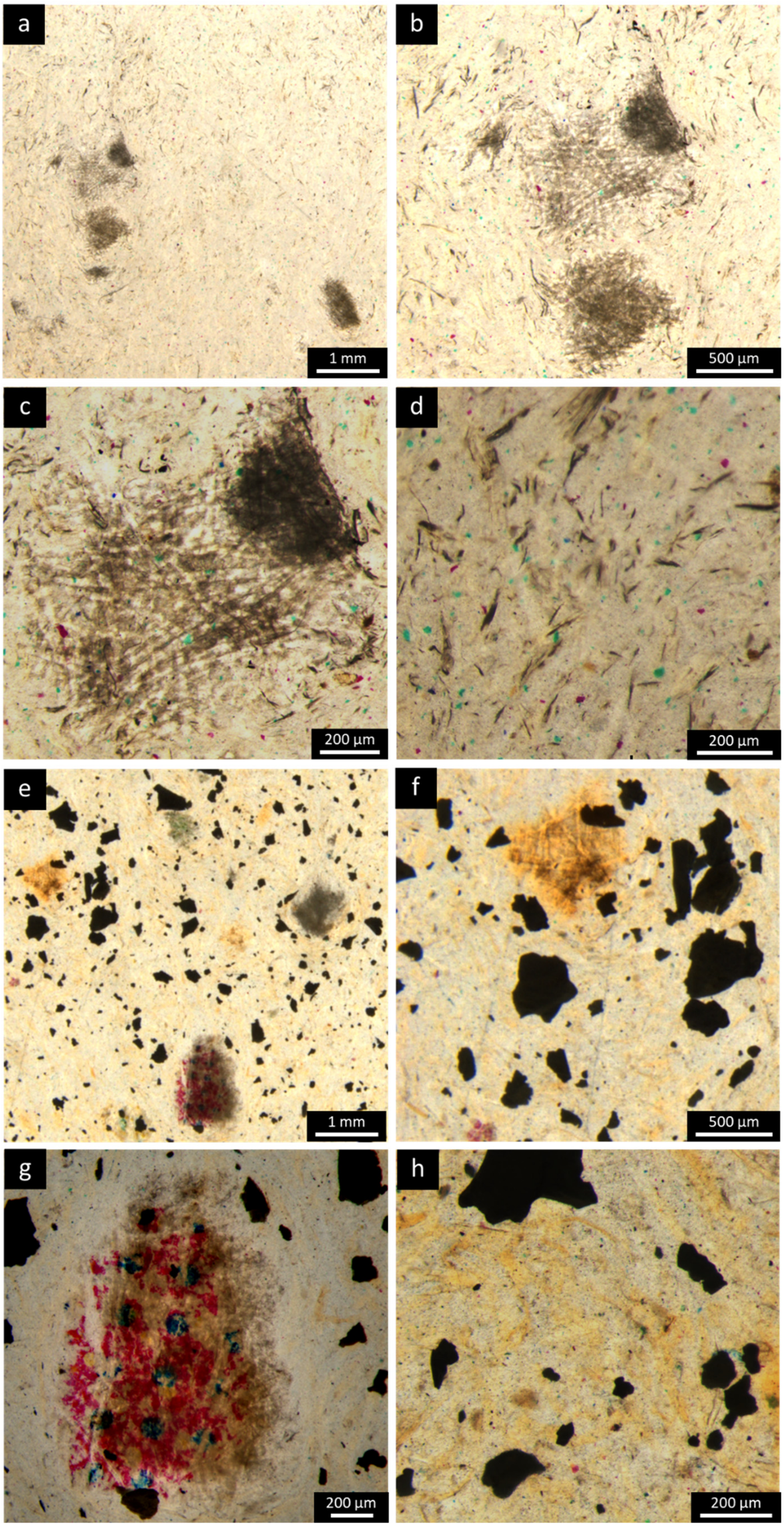

Figure S2. Optical micrographs (transmitted light) of films of the M0 C40 (a-d) and M0 CAl40 (e-h) samples, highlighting the presence of single cellulose fibers, along with residual paper-like aggregates. Dark fragments in Figures 4e – 4h are aluminum foil flakes.

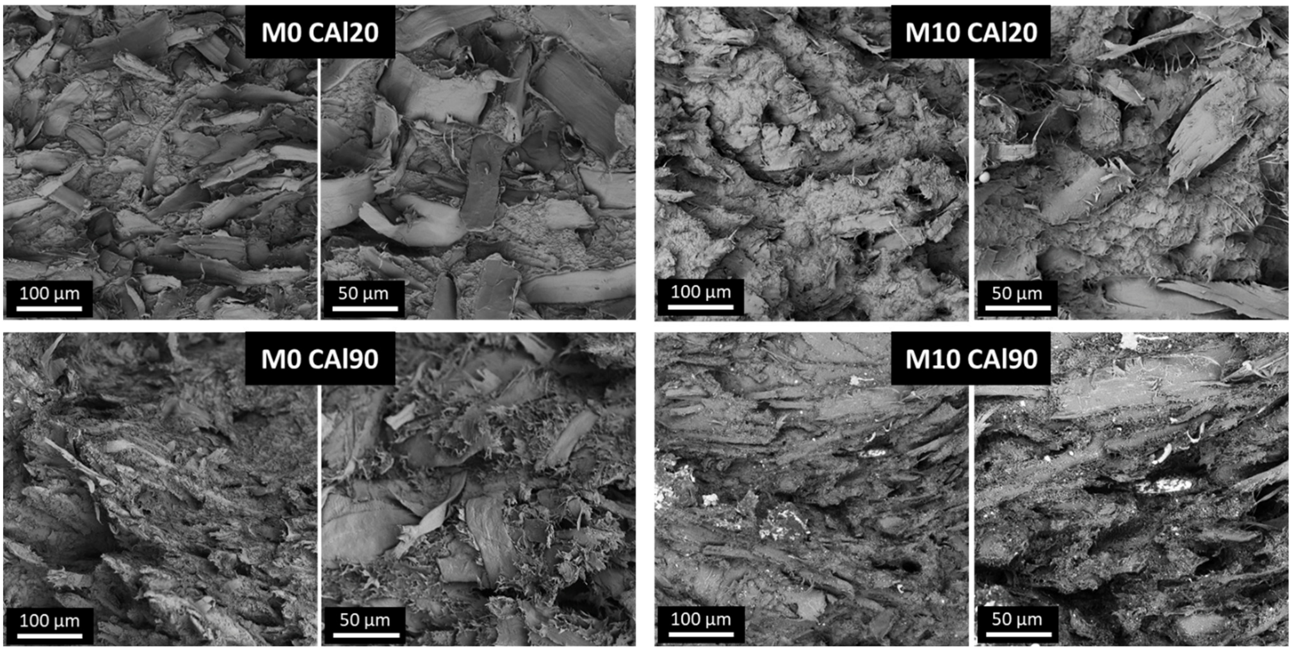

Figure S3. SEM micrographs of impact fracture surfaces of samples containing C-Al cartons, without (M0 CAl20, M0 CAl90) and with (M10 CAl20, M10 CAl90) coupling agent, at different magnification levels.

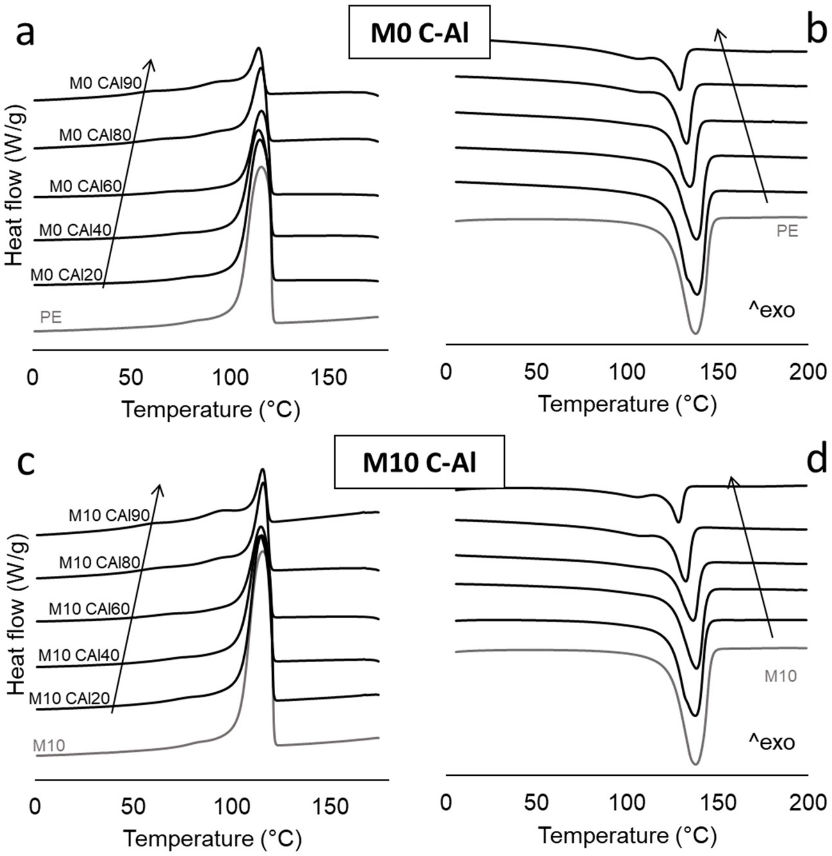

Figure S4. DSC thermograms showing the crystallization and melting peaks of M0 C-Al (a, b) and M10 C-Al (c, d) systems, respectively. Arrows indicate increasing MC content.

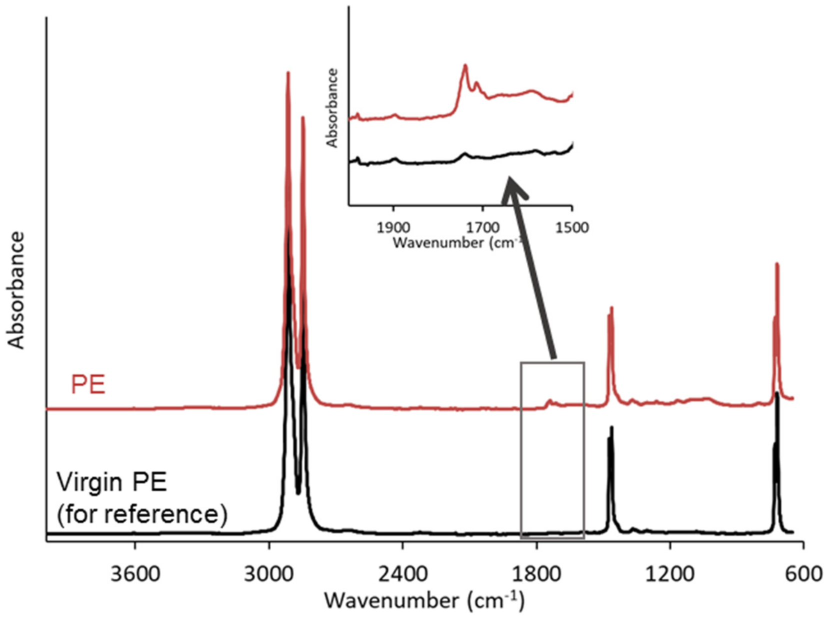

Figure S5. ATR-FTIR spectra of the recycled polyethylene used in this work (PE) compared to a virgin HDPE taken as a reference. In the insert, the carbonyl region is highlighted.

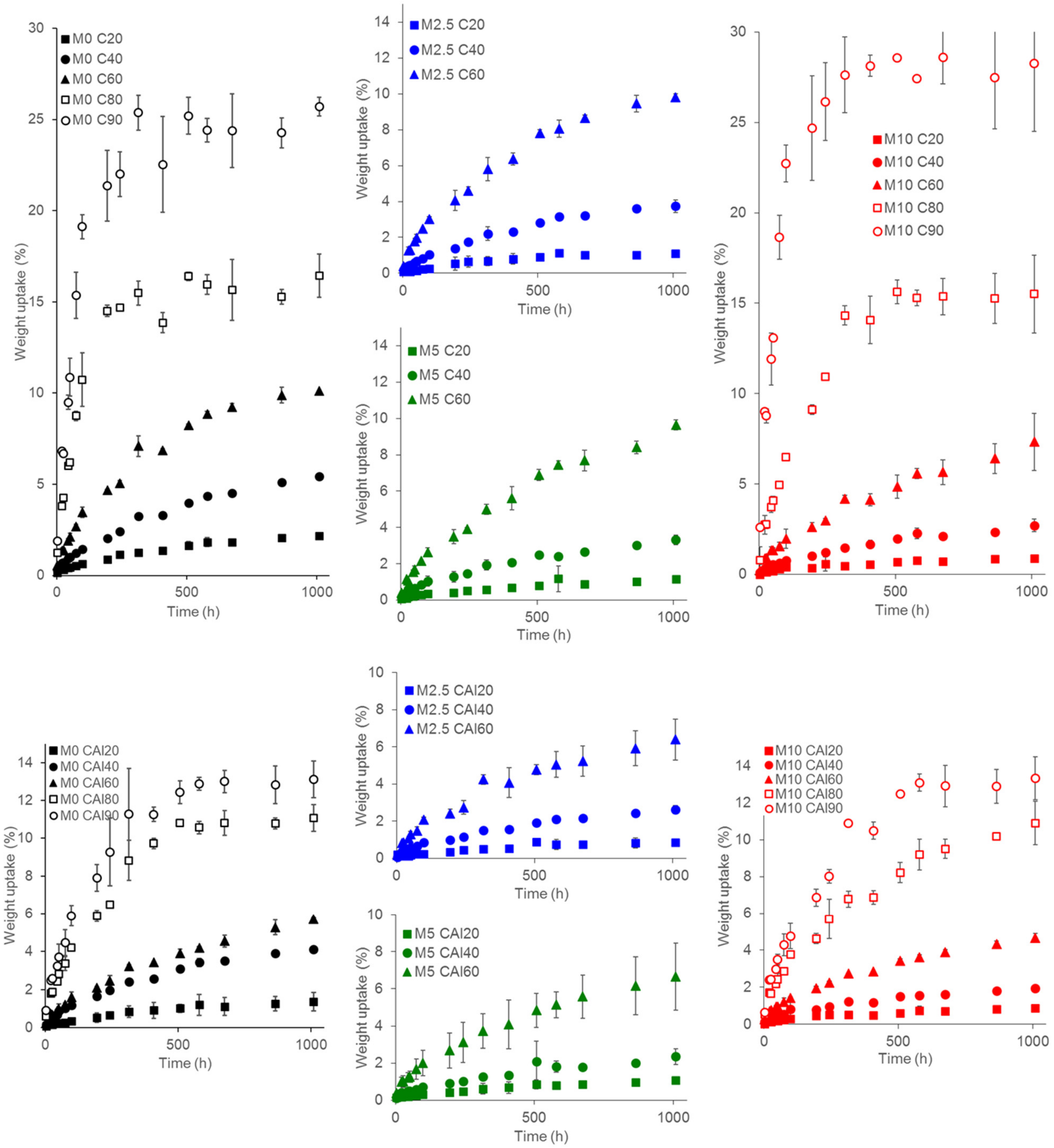

Figure S6. Water absorption as a function of water immersion time for the prepared composites.

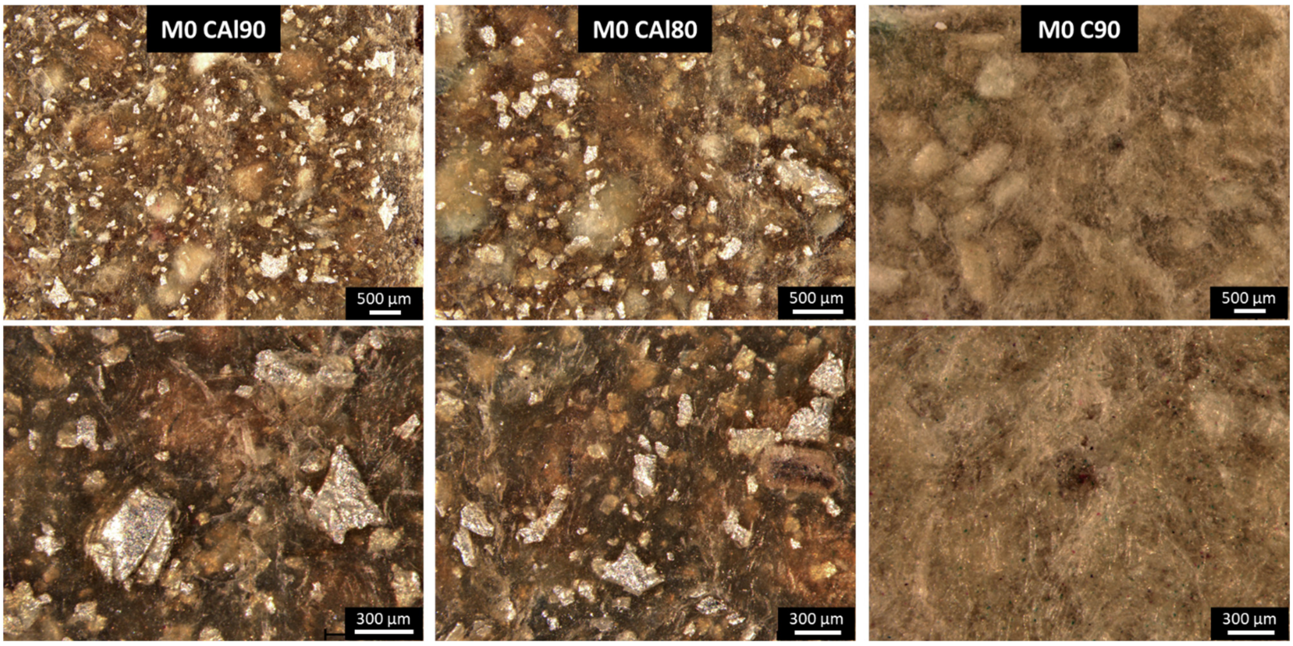

Figure S7. Optical micrographs of the surface of high MC content materials, showing the presence of aluminum foil fragments at the surface in the M0 CAl80, and M0 CAl90 samples.

Techniques.

Details on sieving procedure, SEM, DSC and water absorption analyses are provided in the Materials and Methods section of the paper.

Infrared spectra were collected on a Perkin Elmer Spectrum 100 spectrometer (Perkin Elmer Inc., Whaltam, MA, USA) equipped with a diamond Universal ATR cell. The scanned wavenumber range was 4000–650 cm^{-1}. All spectra were recorded with a resolution of 4 cm^{-1}, averaging 8 scans.

Optical microscopy analysis was carried out by means of a Leica M205C microscope (Leica Camera AG, Wetzlar, Germania).
